# Evaluating temperature-induced regulation of a ROSE-like RNA-thermometer for heterologous rhamnolipid production in *Pseudomonas putida* KT2440

**DOI:** 10.1186/s13568-019-0883-5

**Published:** 2019-09-25

**Authors:** Philipp Noll, Chantal Treinen, Sven Müller, Sabine Senkalla, Lars Lilge, Rudolf Hausmann, Marius Henkel

**Affiliations:** Institute of Food Science and Biotechnology, Department of Bioprocess Engineering (150 k), University of Hohenheim, Fruwirthstr. 12, 70599 Stuttgart, Germany

**Keywords:** Thermoregulation, RNA thermometer, Heterologous rhamnolipid biosynthesis, *Pseudomonas putida*, Biosurfactant

## Abstract

The microbial production of rhamnolipids has been in the focus of research for the last decades. Today, mainly heterologous production systems are targeted due to the advantage of non-pathogenic hosts as well as uncoupling from complex quorum sensing regulatory networks compared to their natural producer *Pseudomonas aeruginosa*. In the recent past, the presence and function of a ROSE-like RNA-thermometer located in the 5′UTR of the rhamnosyltransferase genes *rhlAB* has been reported in wild type *P. aeruginosa*. In this study, the temperature-induced regulation of this native RNA-thermometer for heterologous rhamnolipid production was evaluated and its potential application for process control is discussed. For this purpose, the non-pathogenic production host *P. putida* KT2440 containing the *rhlAB* genes with the native *P. aeruginosa* 5′-UTR region was used. The system was evaluated and characterized regarding the effect of temperature on growth and product formation, as represented by efficiency parameters and yields. Experimental data suggests a major effect of temperature on specific rhamnolipid production rates. With maximum values of 0.23 g/(g h) at 37 °C, this constitutes a more than 60% increase compared to the production rate of 0.14 g/(g h) at the growth optimum of 30 °C. Interestingly however, control experiments unveiled that besides the regulatory effect of the RNA-thermometer, multiple metabolic effects may contribute equally to the observed increase in production rate. As such, this work constitutes an important step towards the utilization of temperature-based process designs and enables the possibility for novel approaches for process control.

## Introduction

Temperature has a direct effect on fundamental biological systems, including enzyme activity and correct folding of proteins. In nature, bacteria experience temperatures that are far from the optimum for growth, and more extreme temperatures can be detrimental for cells. While high temperatures account for denatured and misfolded proteins, low temperatures may cause damage to membranes. Consequently, bacteria have developed different systems to sense changes in environmental conditions such as temperature and induce an adaptation of metabolism and gene expression. Many different principles of thermoregulation have been identified in bacteria, which can be assigned to the class of protein-, DNA- or RNA-thermosensors. The principle of action behind these strategies is the conversion of temperature signals into either transcriptional or translational responses. The different principles have been extensively reviewed (Klinkert and Narberhaus 2009; Shapiro and Cowen 2012; Sengupta and Garrity 2013). Protein-based thermosensors are a very diverse group including transcriptional regulators, sensor kinases, chaperones or proteases (reviewed in Klinkert and Narberhaus 2009). Due to the inherent diversity in the underlying mechanisms, protein-based thermosensors affect different cellular processes such as transcription, translation, protein stability, signal transduction as well as proteolytic processes. Changes in temperature are typically sensed as a result of conformation changes of protein structure as well as misfolded proteins. These thermosensors are part of a regulatory network, such as the production of heat shock proteins mediated by sigma factor 32 (RpoH) in *Escherichia coli* or the transcriptional repressor of heat-shock genes HrcA in *Bacillus subtilis* (Hecker et al. 1996; Narberhaus 1999).

DNA thermosensors, also referred to as DNA thermometer, are AT-rich sequences that alter the structure and bending of DNA in response to temperature. DNA thermometer are known to be present in promoter region where they can affect binding and interaction with regulatory proteins such as transcription factors and RNA polymerase resulting in temperature-dependent transcription (Nickerson and Achberger 1995; Katayama et al. 1999).

The third class of thermosensing is based on the temperature-dependent conformation of specific RNA sequences, which are termed RNA-thermometer (RNAT). These sequences typically include the 5′-untranslated region in mRNA including the ribosome binding site (Shine-Dalgarno sequence) and in some cases the start codon (Klinkert and Narberhaus 2009). The temperature-dependent three-dimensional structure formed by the RNA occludes ribosome binding at low temperatures. At higher temperatures, the structure destabilizes and normal translation is facilitated (Chowdhury et al. 2006). The first cis-encoded temperature-sensitive RNA sequence which regulates translation was discovered in 1989 (Altuvia et al. 1989). Several examples for bacterial genes which are induced by a shift in temperature due to the presence of RNA-thermometer have been reported up to today, and many are linked to heat-shock and virulence associated genes (Nocker et al. 2001; Johansson et al. 2002; Waldminghaus et al. 2007; Kortmann and Narberhaus 2012). Even though most RNA-thermometers have been reported to be present in the 5′-UTR, exceptions like the *rpoH* RNA-thermometer have been reported to include significant parts of the coding region in the transcript. More than 200 nucleotides of the coding region of *rpoH*, divided into two segments, are involved in the temperature response of the transcript. Furthermore, the Shine-Dalgarno sequence in the *rpoH* mRNA is, unlike to the majority of described RNATs, not completely blocked (Morita et al. 1999). One of the most abundant sequences present in the 5′-UTR of many different bacteria controlling heat shock response is referred to as ROSE (repression of heat-shock gene expression) element (Nocker et al. 2001; Balsiger et al. 2004; Wei and Murphy 2016). Besides these simplest single-loop conformations, RNA-thermometers with up to five loops have been reported (Klinkert et al. 2012; Kouse et al. 2013). RNA-thermometer, unlike riboswitches, do not require a ligand to function properly, and the temperature-dependent regulatory function is mediated solely by the sequence of the RNA (Chowdhury et al. 2006; Serganov and Nudler 2013). For pathogenic bacteria, a change of environmental temperature is a key stimulus that may indicate infection of the host. To facilitate efficient growth under these conditions, and as defense against immune response of the host, the expression of virulence factors needs to be closely regulated. In many pathogenic bacteria, expression of virulence genes is induced at 37 °C, and the underlying mechanisms are often based on RNA-thermometers (Johansson et al. 2002; Böhme et al. 2012; Weber et al. 2014). *Pseudomonas aeruginosa* is an opportunistic human pathogen, which is known for infections of the respiratory tract, the eyes as well as the skin.

*Pseudomonas aeruginosa* furthermore is known for the production of rhamnolipid biosurfactants, which are glycolipids consisting of a (hydroxyalkanoyloxy)alkanoic acid (HAA) hydrophobic fatty acid tail and a hydrophilic head of one or two rhamnose molecules. While the expression of virulence associated genes as well as genes involved in rhamnolipid biosynthesis is under control of a complex quorum sensing dependent regulatory network, the effect of RNA-thermometers is not well-understood. The presence of an RNA-thermometer in the *rhlAB* operon coding for the rhamnosyltransferase complex required for rhamnolipid biosynthesis, was first predicted in silico in the past (Waldminghaus et al. 2007), and first experimental evidence of its function was recently provided by Grosso-Becerra et al. (2014). Expression of *rhlA* was shown to be increased at 37 °C, which is reported to be caused by a ROSE-like element in the 5′-UTR. At temperatures ≤ 30 °C, an inhibitory loop structure blocks *rhlA* translation, which subsequently results in a polar effect of *rhlB* transcription that is initiated at the *rhlA* promotor (Grosso-Becerra et al. 2014).

The microbial production of rhamnolipids has been in the focus of research for the last decades. As green alternatives to petrochemically derived surfactants, rhamnolipids have gained more and more attention over the last decades. The relevance of rhamnolipids as products of industrial biotechnology is furthermore emphasized by patents from the last years as well as the recent commercialization by Evonik Industries (Schaffer et al. 2011; Schilling et al. 2013; Evonik Industries 2016). In current research, heterologous production systems are targeted due to the advantage of non-pathogenic hosts as well as uncoupling from complex quorum sensing regulatory networks compared to their natural producer *Pseudomonas aeruginosa*.

Even though the structure and function of the native RNA-thermometer from *P. aeruginosa* PAO1 has been reported in the past, its potential has not yet been evaluated for application in process development and heterologous production processes. Using temperature as a physical inductor poses advantages over chemical inducers used today. Changing temperature is regarded as more cost efficient as opposed to adding chemical inducers (e.g. IPTG). Furthermore, the risk of contamination can be avoided when simply changing the temperature compared to the addition of a chemical inducer to a closed system, which may be beneficial for process validation, such as in the pharmaceutical environment.

**Fig. 1:**
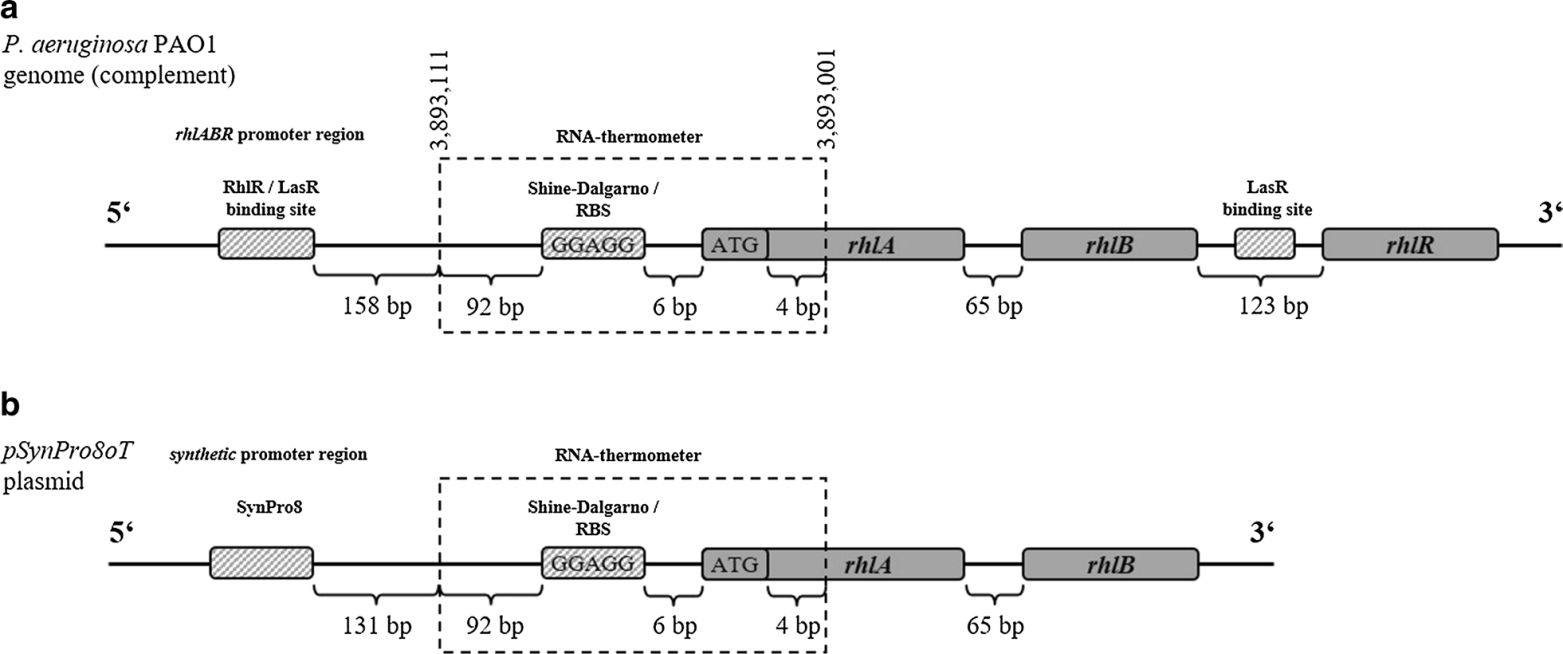
Comparison of DNA sequences of the *rhlAB* operon with a ROSE-like element RNA-thermometer located in the 5′UTR of native (**a**) and heterologous (**b**) rhamnolipid production hosts *P. aeruginosa* PAO1 and *Pseudomonas putida* KT2440 pSynpro8oT_*rhlAB* (Beuker et al. 2016b)

In this study, the temperature response and potential application for process control of this native RNA-thermometer for heterologous rhamnolipid production was evaluated. The plasmid-based system contained the *rhlAB* genes with the native *P. aeruginosa* 5′-UTR region and RNA-thermometer sequence under control of a synthetic promoter as described previously (Beuker et al. 2016b) (Fig. 1). The system was evaluated and characterized regarding the effect of temperature on growth and product formation, as represented by efficiency parameters and yields. As such, this work constitutes a fundamental step toward establishing temperature-based process design and control.

## Materials and methods

### Chemicals and reference substances

All chemicals used in the current study were purchased from Carl Roth GmbH (Karlsruhe, Germany) if not stated otherwise. All chemicals for analytic procedures were of analytical/MS grade. The di-rhamnolipid (Rha-Rha-C_10_-C_10_) standard for HPTLC analysis was a gift from former Hoechst AG (Frankfurt-Hoechst, Germany). The mono-rhamnolipid (Rha-C_10_-C_10_) standard was obtained from Sigma-Aldrich Laborchemikalien GmbH (Seelze, Germany). Rhamnolipid derivatization was performed as described by Schenk et al. (1995), and for this purpose, 4-bromophenacylbromide and triethylamine were obtained from Sigma-Aldrich Laborchemikalien GmbH (Seelze, Germany).

### Microorganism and plasmids

*Pseudomonas* *putida* KT2440 with plasmid pSynPro8oT_*rhlAB* producing mono-rhamnolipids was used as described previously (Beuker et al. 2016b). As control with no RNA-thermometer effect, plasmid pSynPro8oT_*rhlAB*-*oT* which carries the RNA-thermometer sequence inactivated by randomization was constructed. The pBBR1MCS-3 based plasmid pSynPro8oT_*rhlAB* was extracted from *Pseudomonas* *putida* KT2440 pSynPro8oT_*rhlAB.* The template plasmid pSynPro8oT_*rhlAB* was modified in a polymerase chain reaction using a Q5 High-Fidelity Polymerase (NEB, Ipswitch, MA, USA), the forward primer 5′ TGA CTT CAA TTG TTC TGG TTA GTA ATG CTA TGA GTG AAC GGG GGC TGA AGT TGG GAG GTG TGA AAT GCG GC 3′ and the reverse primer 5′ TGA TGT CAA TTG GTC CAT TTG GTT AAT TCA TGT ATG ATC GAG ATA GGC TGA TAA ACT CAG CGG CCA TCC GC 3′. The primers were designed to contain a 5′ overhang of 6 bp, a MfeI restriction site to produce sticky ends facilitating later religation with T4 DNA Ligase of the linearized vector containing the randomized sequence. The randomized sequence is of equal length like the to be exchanged RNAT. The plasmid containing the randomized sequence instead of the RNAT was transformed into electro competent *Pseudomonas* *putida* KT2440 wild type cells. Electro competent *Pseudomonas* *putida* KT2440 wt were prepared according to Choi et al. (2006). The solution containing the PCR product (NoRNAT) was desalted using a 0.025 µm nitrocellulose membrane. Electroporation of electro competent cells was performed using an electroporation device (Eporator, Eppendorf AG, Hamburg, Germany) with voltage set to 2.5 kV. After transformation cells were transferred to 1 mL LB-Medium and incubated for 3 h at 30 °C and 120 rpm. Consequently, cells were plated on LB-Agar containing 20 mg/L Tetracycline as selection marker and incubated overnight at 30 °C. After streaking on LB-Agar containing 20 mg/L Tetracycline an isolated single colony was selected for preparation of glycerol stocks, cultivation and sequence confirmation of *Pseudomonas* *putida* KT2440 pSynPro8oT_*rhlAB*-*oT*.

### Media preparation

All growth media (LB, ModR, SupM) were prepared as described by Beuker et al. (2016a).

### Cultivation conditions

All shake flasks were inoculated in a shake incubator (New Brunswick™/Innova^®^ 44, chamber, Eppendorf AG, Hamburg, Germany) at 120 rpm and a temperature between 26 and 40 °C, as indicated for each individual experiment. First precultures were prepared by inoculating 25 mL LB medium in a 250 mL baffled shake flask with 50 µL from a glycerol stock solution of *P.* *putida* KT2440 pSynPro8oT_*rhlAB and* pSynPro8oT_*rhlAB*-*oT* and incubation at 30 °C for 24 h. From these first precultures, grown for 24 h, seed cultures were prepared for all preformed experiments in shake flasks as described by Beuker et al. (2016a). For each seed culture, grown for 15 h, 1 mL of the first preculture was used to inoculate 100 mL SupM medium in 1 L shake flasks. The main culture in ModR medium was inoculated with the second preculture to an OD_600_ of 0.3.

### Sampling and sample processing

Sample processing was performed as described by Beuker et al. (2016a). For extraction of rhamnolipids, culture supernatant was acidified 1:100 (*v*/*v*) with phosphoric acid and extracted twice with 1.25:1 (*v*/*v*) ethyl acetate, pooled and evaporated in a vacuum centrifuge at 40 °C for 40 min and 10 mbar (RVC 2-25 Cdplus, Martin Christ Gefriertrocknungsanlagen GmbH, Osterode am Harz, Germany).

### HPTLC for quantification of rhamnolipids

Rhamnolipid detection was performed as described by Schenk et al. (1995) with modifications for a protocol suitable for HPTLC (Horlamus et al. 2018). Evaporated rhamnolipid samples were dissolved in acetonitrile and derivatized for 90 min at 1400 rpm and 60 °C using a 1:1 mixture of 135 mM bromphenacylbromid and 67.5 mM tri-ethyl-ammonium/-amin as described by Cooper and Anders (1974). If Rhamnolipid concentrations above 2 g/L were to be expected samples were diluted prior to derivatization. Measurements of derivatized samples were performed on silica gel 60 HPTLC plates with fluorescence marker F^254^ (Merck, Darmstadt, Germany). A HPTLC system for quantitative analysis (CAMAG Chemie-Erzeugnisse & Adsorptionstechnik AG, Muttenz, Switzerland) was used. Samples were applied with the Automatic TLC Sampler 4 (ATS 4). Development of plates was performed with the automatic Developing Chamber 2 (ADC 2) equipped with a 20 cm × 10 cm twin-trough chamber. After development, plates were analyzed using the TLC Scanner 4. The HPTLC system is controlled via an HPTLC imaging and data analysis software (winCATS 1.4.7.2018 software, CAMAG, Muttenz, Switzerland). For sample application the filling speed of the syringe was adjusted to 15 µL/s and dosage speed to 150 nL/s. In between sample application the syringe was rinsed with methanol. Band width was adjusted to 6 mm and application start was at 15 mm from the left and 8 mm from the lower edge. Mobile phase for plate development was composed of 30:5:2.5:1 isopropyl acetate:ethanol:water:acetic acid. Tank saturation was adjusted to 5 min and drying with an air stream was carried out for 5 min afterwards. A deuterium (D2) lamp with slit dimensions of 3 mm × 0.3 mm was used for scanning. Scanning was performed at 263 nm at which wavelength bromphenacyl-derivatized rhamnolipid congeners absorb. Data resolution and scanning speed were set to 1 nm/step and 100 nm/s respectively.

### Enzymatic assays

The concentration of glucose was detected from the culture supernatant of samples using glucose enzymatic assay kits (R-Biopharm AG, Darmstadt, Germany), according to the manufacturers’ instructions.

### Software for graphical analysis, regression and replicates

Regression analysis of measured data was performed using scientific graphing and data analysis software (SigmaPlot, Systat Software Inc., San Jose, CA). Specific rhamnolipid production rates and growth rates were calculated using a four-parameter logistic fit for biomass and rhamnolipid concentration (Henkel et al. 2014). All data was obtained as duplicates from at least two independent biological experiments, and measurement results are presented as mean ± standard deviation.

### Software for parameter optimization, modeling and simulation

All parameter fitting and simulation for temperature-dependency models, as described in “Results” section, was performed in MATLAB (The MathWorks, Natick, MA, USA). For parameter optimization, the Nelder-Mead numerical algorithm implemented in the embedded functions “fmincon” and “fminsearch” by minimizing the error of simulation data and measured data according to a least-square error function was applied. To visualize and describe temperature dependency of rhamnolipid production rates, a modified Ratkowsky model according to Zwietering et al. (1991; Ratkowsky et al. 1983) was used to derive a fitting curve.

1 $$q_{RL} \left( T \right) = \frac{{\left[ {b \cdot \left( {T - T_{min} } \right)} \right]^{2} \cdot \left\{ {1 - e^{{c \cdot \left( {T - T_{max} } \right)}} } \right\}}}{a}$$

For the maximum specific growth rate µ_max_, a fitting curve was derived using a model proposed by Roels et al. (1983).

2 $$\mu_{max} \left( T \right) = \frac{{A \cdot e^{{\left( {\frac{{ - E_{G} }}{RT}} \right)}} }}{{1 + B \cdot e^{{\left( {\frac{{ - \Delta G_{d} }}{RT}} \right)}} }}$$

## Results

**Fig. 2:**
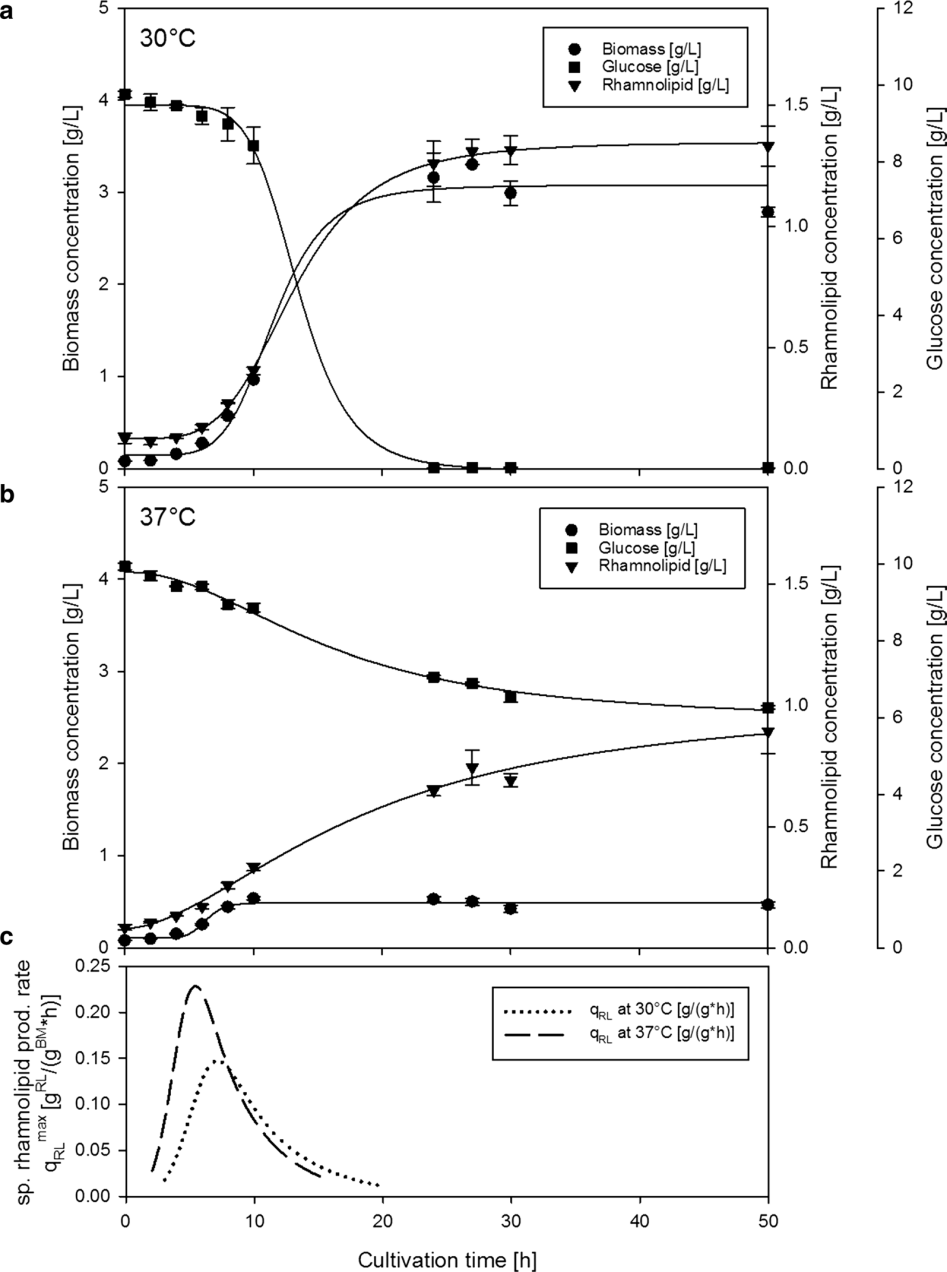
Time course of biomass concentration (circles), rhamnolipid concentration (triangles) and glucose concentration (squares) during shaking flask cultivation of *Pseudomonas putida* KT2440 pSynpro8oT_*rhlAB* on ModR medium with 10 g/L glucose at 30 °C (**a**) and 37 °C (**b**), and corresponding course of specific rhamnolipid production rate (**c**)

To visualize the effect of temperature on rhamnolipid production and growth, a first experiment was performed comparing cultivations at 30 °C (growth optimum, Fig. 2a) and 37 °C (suggested induction, Fig. 2b). There was no apparent lag phase detected in either cultivation, but a significantly lower biomass concentration was achieved at 37 °C of 0.6 g/L, while 3 g/L were obtained at 30 °C. Even though significantly lower biomass concentrations (approx. 1/5th) were produced at 37 °C, the maximum rhamnolipid concentration was only slightly lower when comparing 30 °C (1.2 g/L) and 37 °C (0.9 g/L). Visualization of this effect is further supported by plotting of specific rhamnolipid production rates over the course of cultivation (Fig. 2c) for 30 °C and 37 °C, which reveals approx. 60% increased specific rhamnolipid productivity from 0.14 g/(g h) to 0.23 g/(g h).

**Fig. 3:**
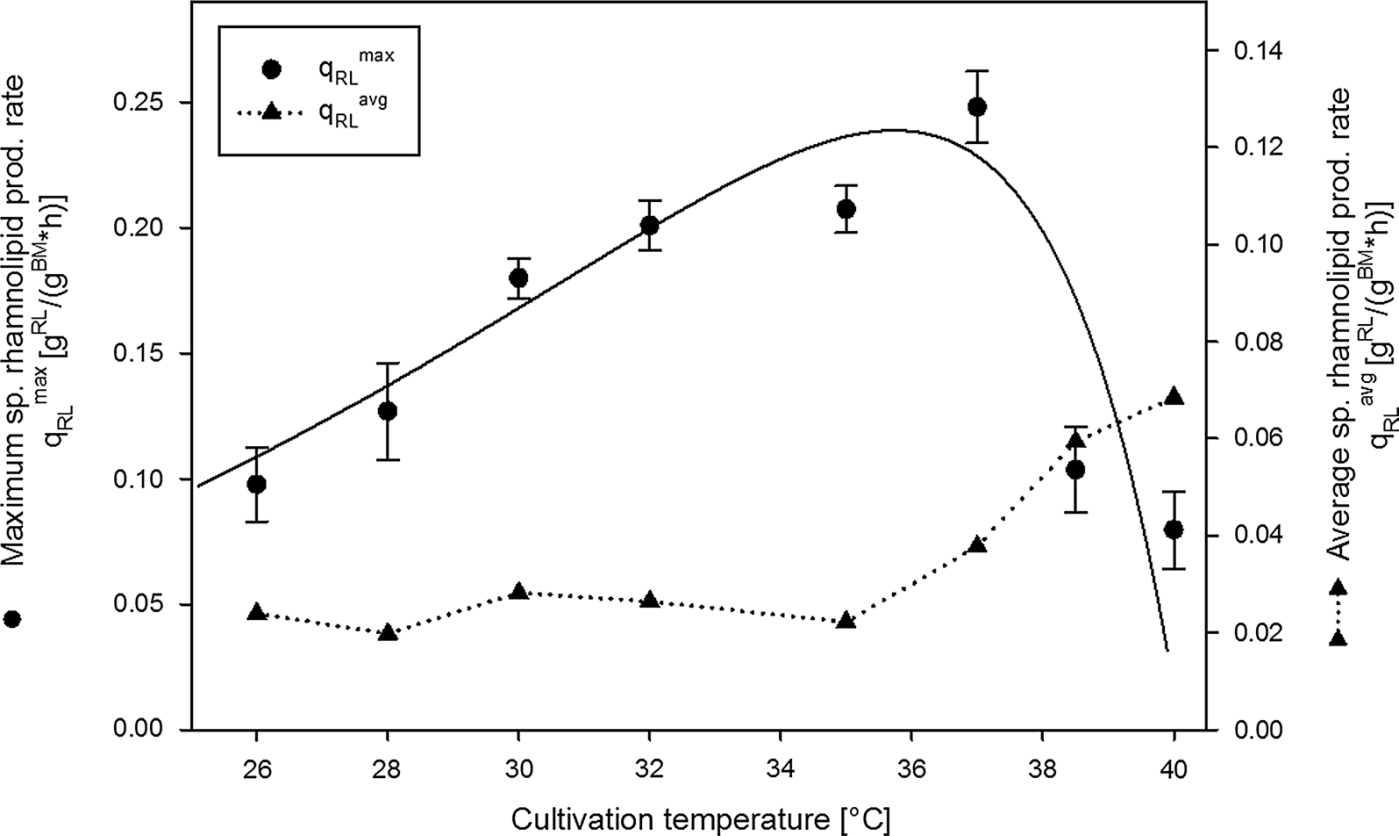
Maximum (circles) and average (triangles) specific rhamnolipid production rates at different cultivation temperatures. Maximum specific rhamnolipid production rates as a function of temperature was described using the empirical modified Ratkowsky equation (Eq. 1) (Ratkowsky et al. 1983; Zwietering et al. 1991), with parameter fitting performed for the obtained data (black line)

This temperature-dependency of rhamnolipid production (Fig. 2) was further investigated at temperatures between 26 and 40 °C and is shown in Fig. 3. As a fitting curve for the maximum specific rhamnolipid production rate, a modified Ratkowsky model (Eq. 1) according to Zwietering et al. (1991) was used, as described in “Materials and methods”. The productivity increased in a linear way until a maximum of 0.23 g/(g h) was reached, which, according to the model with parameter fitting performed for the obtained data, is slightly above 36 °C or, when comparing experimental data, 0.24 g/(g h) at 37 °C. At temperatures above 37 °C, rhamnolipid productivity decreased rapidly. When comparing the average sp. productivity to the maximum sp. productivity, the average productivity was unaffected and constant at a value of approximately 0.05 g/(g h) at temperatures between 26 °C and approximately 35 °C. At temperatures above 35 °C however, the average productivity increased nearly threefold to values of 0.14 g/(g h).

**Fig. 4:**
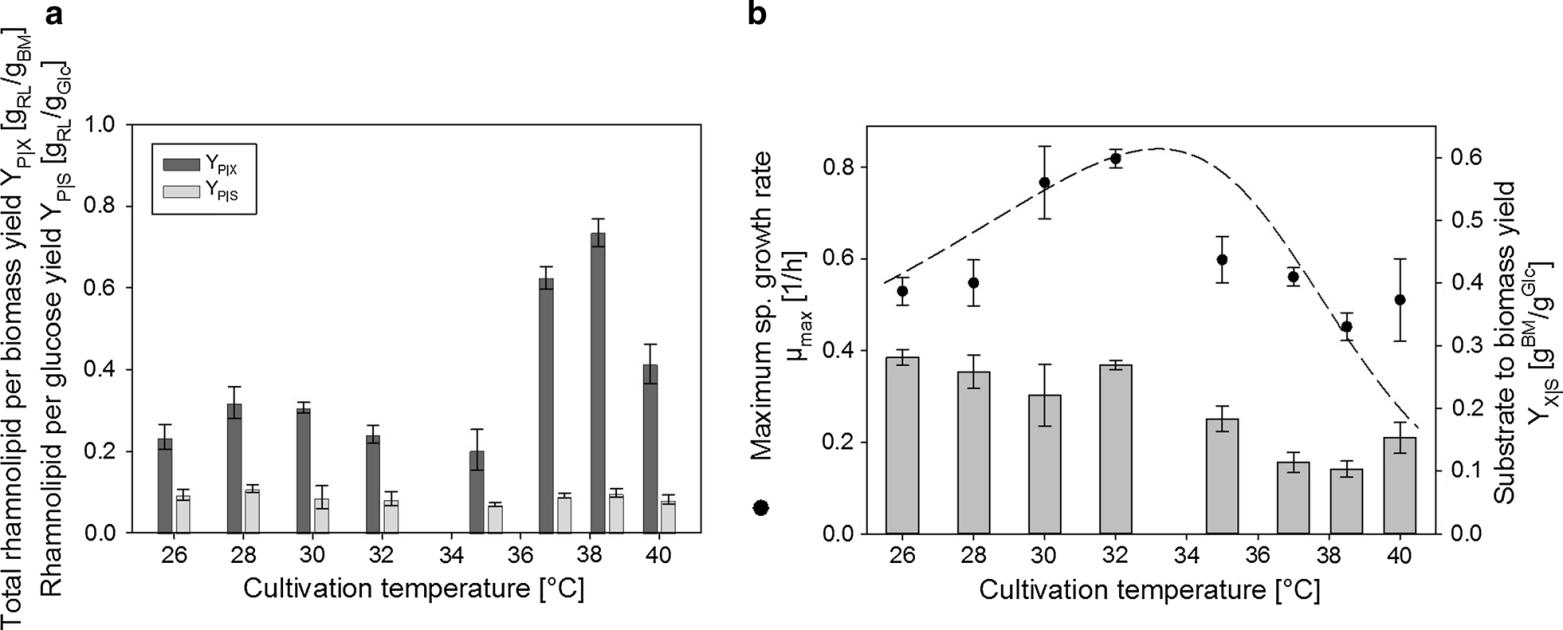
Effect of temperature on rhamnolipid-per-glucose yields (Y_P|S_) and rhamnolipid-per-biomass yields (Y_P|X_) (**a**) as well as effect of temperature on maximum specific growth rate *µ*_max_ and substrate-to-biomass yields (Y_X|S_) (**b**). Maximum specific growth rate as a function of temperature was described using an equation proposed by Roels et al. (1983) (Eq. 2) with parameter fitting performed for the obtained data (dashed line)

Rhamnolipid-per-glucose yields (Y_P|S_) and total rhamnolipid-per-biomass yields (Y_P|X_) are shown for different temperatures in Fig. 4a. There was no apparent correlation of rhamnolipid-per-glucose yield and temperature and Y_P|S_ stayed almost constant at values of 0.06–0.08 g/g over the entire investigated temperature range. The total rhamnolipid-per-biomass yield was also almost unaffected by temperature between 26 °C and approximately 35 °C. At temperatures above 35 °C however, Y_P|X_ reached values of up to 0.75 g/g at 38.5 °C, which were approximately 2.5-fold increased when comparing to Y_P|X_ at lower temperatures. At 40 °C however, this highest value decreased significantly to approx. 0.4 g/g (Fig. 4a).

In addition to rhamnolipid production rates and yields, growth rates and substrate-to-biomass were furthermore investigated for all cultivations (Fig. 4b). Data reveals that the optimum temperature for growth was at approximately 30–32 °C. The maximum specific growth rate µ_max_ = 0.7–0.8 1/h was determined by a fitting curve of a temperature dependent equation (Eq. 2) according to Roels et al. (1983). While glucose-to-biomass yields (Y_X|S_) were relatively constant at values of 0.35–0.40 g/g at the growth optimum and temperatures below, there was a decrease in yield at temperatures above 32 °C to below 0.2 g/g (Fig. 4b).

**Fig. 5:**
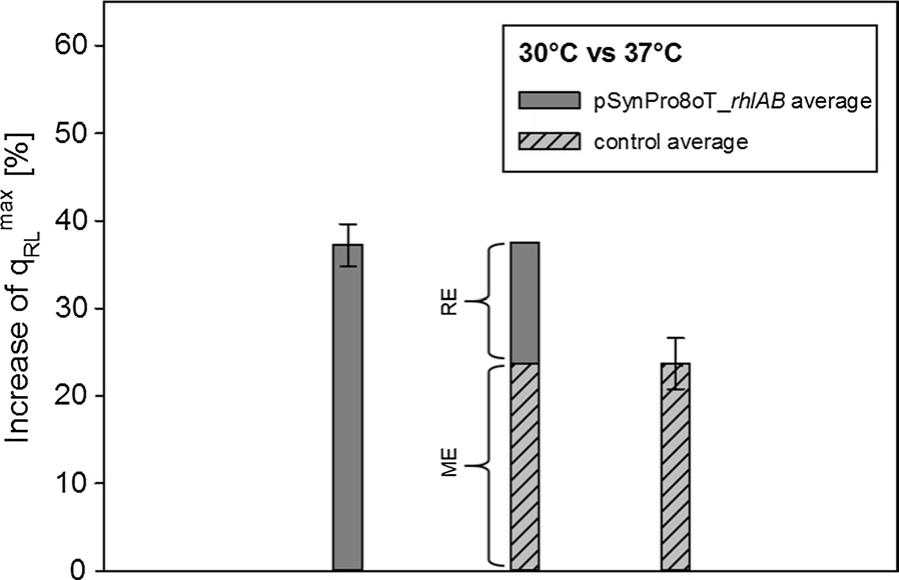
Comparison of increase in specific rhamnolipid production rate of *Pseudomonas putida* KT2440 pSynpro8oT_*rhlAB* and control plasmid with inactivated RNA-thermometer at 30 °C and 37 °C respectively. Average differences in production rate calculated from individual biological data are shown with derived metabolic effect (ME) and regulatory (RE) effect

As a control, a plasmid with inactivated RNAT was used to determine the increase in specific rhamnolipid production rate by performing comparing cultivations with both constructs (Fig. 5). For *P. putida* KT2440 pSynpro8oT_*rhlAB*, an average increase in specific productivity of approx. 36% was measured when comparing cultivations at 30 °C and 37 °C, respectively (see also Fig. 3). For cultivations of *P. putida* KT2440 including the plasmid with randomized and inactivated RNA-thermometer (open conformation) at 30 °C and 37 °C, an average increase in specific rhamnolipid production rate of approx. 23% was detected.

## Discussion

When comparing the time courses of biomass concentration during cultivations at 30 °C to 37 °C, final biomass at 37 °C was significantly lower while the final rhamnolipid concentration remained nearly comparable to what was obtained at 30 °C (Fig. 2a, b). Therefore, it can be reasoned that a higher biomass specific productivity was reached at 37 °C. After calculating specific productivity over time using data from continuous curve fits (Fig. 2c), a similarly shaped time course of productivities was observed, and the main difference between 30 °C and 37 °C was the overall extension and height (Fig. 2c). This suggests that indeed, specific production rates are higher at a cultivation temperature of 37 °C instead of being attributed to other phenomena such as increased duration of the productive phase.

As a next step, the temperature-dependency of rhamnolipid production was investigated in a range between 26 and 40 °C (Fig. 3). An almost linear increase of rhamnolipid productivity between 26 °C until the optimum at 37–38 °C could be observed in the recorded temperature range of 26–40 °C. Assuming rhamnosyltransferase concentration and rhamnolipid production rate to be proportional in the absence of limitations, this correlation mirrors what is known on RNA-thermometers regulation behavior. It is reported that the RNA loop typically never completely prevents translation but shows a gradually increasing temperature-response profile when thermal energy is introduced into the system because of the destabilizing effect of elevated temperatures on the inhibitory loop structure (Chowdhury et al. 2006; Rinnenthal et al. 2010). After reaching the optimal rhamnolipid production temperature, productivities rapidly decrease when temperatures are further increased. This may be due to different effects elevated temperatures can have on bacteria. Furthermore, possible indirect effects on rhamnolipid formation have to be considered, which are due to altered bacterial metabolism resulting in a decreased precursor concentration causing a bottleneck for rhamnolipid production. Rhamnolipid synthesis itself may be negatively affected by temperatures above the optimal production temperature by defective or altered function or substrate binding capacity of RhlA respectively RhlB towards the precursors HAA and dTDP-rhamnose.

When comparing how the average specific rhamnolipid production rate is effected by cultivation temperature (Fig. 3), interestingly, an increase is only detected once temperature of highest rhamnolipid production is surpassed. The average specific rhamnolipid production rate may represent an important target parameter for optimization for evaluation of the total productivity of the process. It is important to note, however, that the average specific rhamnolipid production rate is calculated from shake-flask with a defined and limited cultivation time. In contrast this may not be the case for optimized prolonged processes with a fed-batch design for instance. Total rhamnolipid yield per biomass or glucose moreover represent important target optimization parameters for bulk chemical production processes. In terms of development of an economical process, it should be noted that observed increase in rhamnolipid yield per biomass (Fig. 4a) correlates with the maximum specific production rate (Fig. 3). This would generally allow for a process design with both increased as well as more efficient rhamnolipid production.

To determine what proportion of the observed effect of increase in rhamnolipid production can be attributed to a regulatory effect, control experiments using a plasmid with randomized, inactive sequence of the RNA-thermometer (open conformation) were performed. The increase in specific rhamnolipid production rate comparing cultivations of 30 °C to 37 °C of *P. putida* KT2440 pSynpro8oT_*rhlAB* with functional RNAT was quantified to be approx. 36% (Fig. 5, see also Fig. 3). This increase may be assigned to two different (overlapping) effects. First a regulatory effect caused by the inhibitory structure of the RNAT and second by multiple (overlapping) metabolic effects. When assuming no regulatory effect by the RNA-thermometer, the increase for the control strain (with inactivated RNAT) would be expected to be approx. equal to the increase in specific rhamnolipid production rate of the strain with functional RNAT. Metabolic effects would then solely cause this increase. When assuming no overlapping metabolic effects, and increases in production rate were assumed to be fully attributed to the RNA-thermometer regulation, the increase in specific rhamnolipid production rate of the control strain should be close to 0% due to an inactivated RNAT.

Interestingly, control experiments unveiled that besides the regulatory effect (‘RE’, Fig. 5) of the RNA-thermometer, a major part of the increase in specific rhamnolipid production rate is not due to regulation of the RNA-thermometer. The average increase in specific production rate with the control plasmid of around 23% can be attributed to metabolic effects (‘ME’, Fig. 5). However, these results confirm the action of the employed ROSE-like temperature-sensitive regulatory element in a heterologous host, as results obtained with the RNAT plasmid lead to a higher average increase of 36% (Fig. 5).

As temperature has a broad effect on bacterial metabolism and regulation, different hypothesis may provide an explanation for this observed behavior. Firstly, it should be mentioned that RNA-thermometers are generally reported to display a comparably high degree of “leakiness” and inefficiency when inhibiting translation (Chowdhury et al. 2003), which could result in a leveling out of differences in production rates. Furthermore, temperature has an effect on plasmid replication and copy numbers, and therefore also on the number of gene copies present per bacterial cell. Along with effects of increasing temperature on plasmid replication, there are furthermore physical effects that need to be considered, such as a shift in oxygen solubility and therefore availability comparing cultivation temperatures of 30 °C and 37 °C.

Altogether, in this study, the RNA-thermometer effect of a ROSE-like regulatory element could be confirmed in the heterologous host *P. putida* KT2440. Findings from this study along with quantitative data from comparison of efficiency parameters and yields provides a basis of how temperature effects rhamnolipid production and growth. A high potential for using temperature to design and control a process for rhamnolipid production is revealed by the observed strong increase in production rate of up to 60%. Interestingly however, regulation of translation due the RNA-thermometer does not seem to play the major part in the observed effect. As temperature effects a wide range of biological and physical properties, it is difficult to attribute to what extent each individual effect constitutes a part in this effect. Future work could include molecular data such as transcriptome and metabolome data, which would allow insight into bottlenecks, existing effects and limitations during protein biosynthesis. Furthermore, a variety of regulatory RNAT structures are available and may be exploited for process design and control in future studies. RNAT consist of a comparably straightforward structure and mechanism. Therefore, attempts have been made to provide guidelines for in silico design, experimental benchmarking and optimization of synthetic thermosensitive nucleic acids, a procedure that takes only 2–3 weeks claimed by Neupert et al. (2008). In 2017, a toolbox was presented to design RNA-thermometers that offer various temperature responses. The RNATs were designed by Sen et al. (2017) to differ in sensitivity and threshold. The design of the RNAT toolbox for various temperature responses was based on thermodynamic calculations like minimum free energy structures or melting profiles, as well as on experimental activity benchmarking in vitro. A wide range of structures has been found and evaluated to give a varying temperature response within a range between 29 and 37 °C. In the here presented study, even though individual effects are not yet clear, a proof-of-concept for a straightforward method to efficiently increase rhamnolipid production levels is presented. As such, this work constitutes an important step towards the utilization of temperature-based process designs and enables the possibility for novel approaches for process control.

## Data Availability

All discussed data have been included into the manuscript. Please turn to the corresponding author for all other requests.

## Abbreviations

RNAT: RNA-thermometer
ROSE: repression of heat shock gene expression (element)
RL: rhamnolipid
UTR: untranslated region
HPTLC: high-performance thin layer chromatography
HAA: (hydroxyalkanoyloxy)alkanoic acid
